# High *BRCA1* gene expression increases the risk of early distant metastasis in ER^+^ breast cancers

**DOI:** 10.1038/s41598-021-03471-w

**Published:** 2022-01-07

**Authors:** Hui-Ju Chang, Ueng-Cheng Yang, Mei-Yu Lai, Chen-Hsin Chen, Yang-Cheng Fann

**Affiliations:** 1 Institute of Biomedical Informatics, National Yang Ming Chiao Tung University, No. 155, Sec. 2, Li-Nong Street, Taipei, 11221 Taiwan, ROC; 2 Center for Systems and Synthetic Biology, National Yang Ming Chiao Tung University, No. 155, Sec. 2, Li-Nong Street, Taipei, 11221 Taiwan, ROC; 3 Institute of Statistical Science, Academia Sinica, 128 Academia Road, Section 2, Taipei, 115201 Taiwan, ROC; 4 Graduate Institute of Epidemiology and Prevention Medicine, National Taiwan University, 17 Hsu-Chow Road, Taipei, 100025 Taiwan, ROC; 5 Division of Intramural Research, National Institute of Neurological Disorders and Stroke, National Institutes of Health, 35 Convent Dr., Bethesda, MD 20892 USA

**Keywords:** Cancer, Breast cancer, Epidemiology

## Abstract

Although the function of the *BRCA1* gene has been extensively studied, the relationship between *BRCA1* gene expression and tumor aggressiveness remains controversial in sporadic breast cancers. Because the BRCA1 protein is known to regulate estrogen signaling, we selected microarray data of ER^+^ breast cancers from the GEO public repository to resolve previous conflicting findings. The *BRCA1* gene expression level in highly proliferative luminal B tumors was shown to be higher than that in luminal A tumors. Survival analysis using a cure model indicated that patients of early ER^+^ breast cancers with high *BRCA1* expression developed rapid distant metastasis. In addition, the proliferation marker genes *MKI67* and *PCNA*, which are characteristic of aggressive tumors, were also highly expressed in patients with high *BRCA1* expression. The associations among high *BRCA1* expression, high proliferation marker expression, and high risk of distant metastasis emerged in independent datasets, regardless of tamoxifen treatment. Tamoxifen therapy could improve the metastasis-free fraction of high *BRCA1* expression patients. Our findings link *BRCA1* expression with proliferation and possibly distant metastasis via the ER signaling pathway. We propose a testable hypothesis based on these consistent results and offer an interpretation for our reported associations.

## Introduction

Breast cancer is the most common and the leading cause of cancer deaths in women worldwide^1^. The driver genes in hereditary breast cancers include *BRCA1* and *BRCA2.* The BRCA1 protein is well known for its DNA damage repair function^2–4^ and plays a role as a tumor suppressor. Women with a harmful *BRCA1* mutation, which leads to loss of BRCA1 function, commonly develop cancers in hormonal responsive organs^5–7^, such as breasts and ovaries. In addition to DNA damage repair, the BRCA1 protein has many other functions. There is evidence that functional BRCA1 is needed for *ESR1* (gene of estrogen receptor α) transcription^8^. Therefore, *BRCA1*-mutant breast cancers are mostly negative for estrogen receptors^9,10^. In contrast, most sporadic breast cancers are ER^+^ because their BRCA1 genes are usually intact. After tumor formation, BRCA1 regulates mammary cell proliferation by interacting with the estrogen-estrogen receptor (E-ER) signaling pathway^11–13^ synergistically. Therefore, BRCA1 protein is tightly associated with patient survival, not only in hereditary but also in sporadic breast cancers^14–16^.

Approximately two out of three sporadic breast cancers are positive for estrogen receptors. Estrogen may bind to and activate ERs. ERs in turn promote the transcription of downstream genes, such as c-myc^17,18^ and cyclin D1^19,20^, which are involved in proliferation or cell cycle regulation. Therefore, antiestrogen therapy, such as tamoxifen treatment^21^, has become the gold standard for treating ER^+^ breast cancers in all stages. The BRCA1 protein, through direct or indirect interaction, inhibits ER proliferative functions^12,22,23^. Loss of BRCA1 often leads to uncontrolled ER activity and consequently induces breast cancer formation or progression^24–27^. In fact, decreased BRCA1 protein staining is commonly observed in sporadic breast cancers^24,28^ and this phenomenon is associated with high histologic grades^29,30^ and short disease-free survival^16^. Decreased BRCA1 protein has also been reported to confer tamoxifen resistance^31^. On the other hand, tumors^32,33^ were shown to have upregulated *BRCA1* mRNA levels. Moreover, rapid metastasis^34^ was shown to be associated with high *BRCA1* gene expression. One way to explain the protein function loss and high *BRCA1* expression in tumor cells is the autoregulation of the *BRCA1* gene by its own gene product^35^. However, some earlier studies have presented opposite findings^25,36^. Resolving these conflicting observations may shed light on the possibility of using *BRCA1* gene expression as a prognostic predictor.

## Results

In this study, we collected microarray gene expression data from the Gene Expression Omnibus (GEO) repository^37^ (see “Materials and methods” for details). Molecular subtypes and distant metastasis-free survival (DMFS) time were used to assess the aggressiveness of the tumors. Among all the different molecular subtypes of breast cancers, only luminal A and B subtypes were selected to compare *BRCA1* mRNA levels because both subtypes are ER^+^.

### High ***BRCA1*** gene expression is associated with cell proliferation in ER^+^ breast tumors

**Figure 1:**
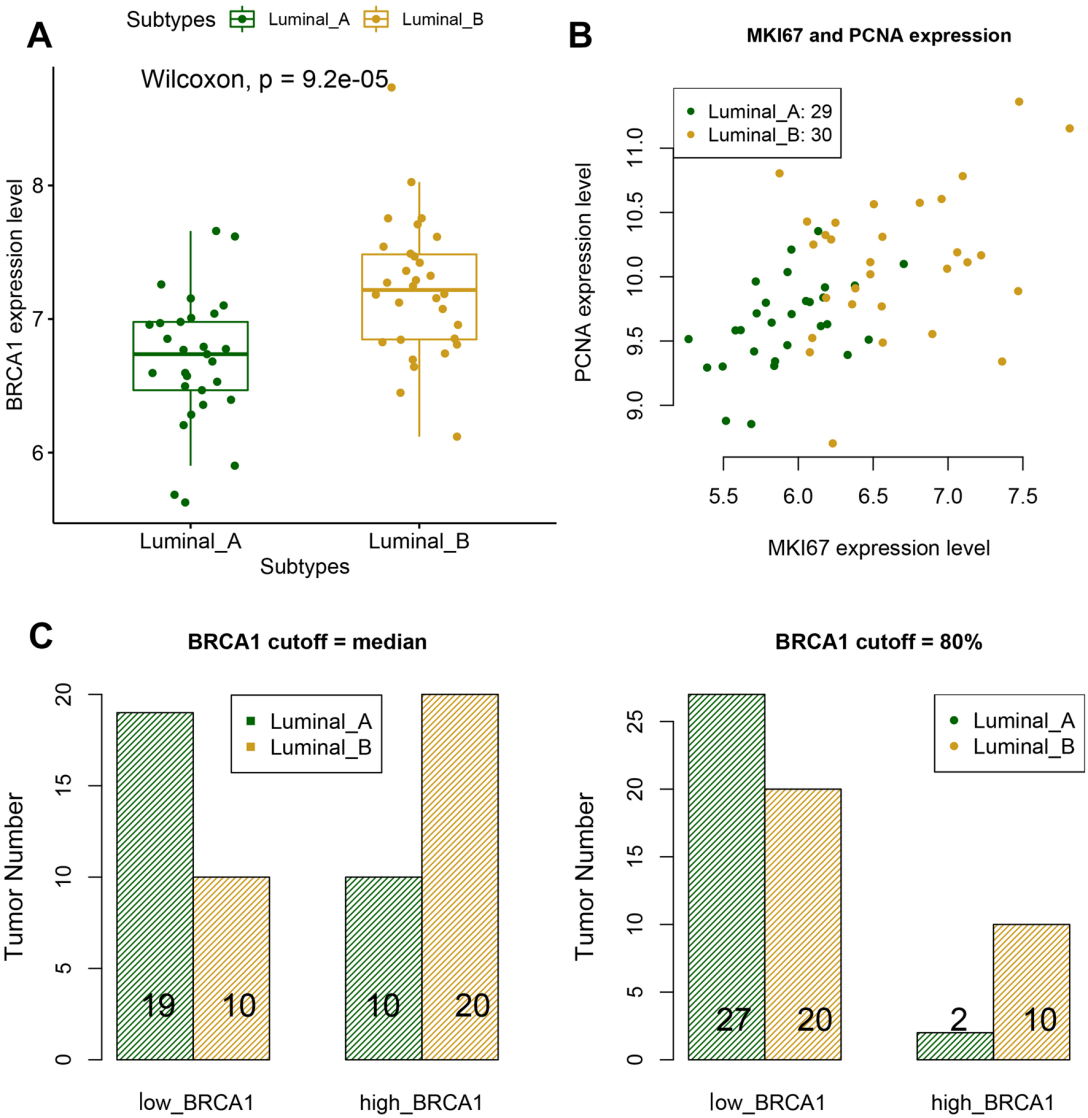
Luminal tumors in dataset GSE45827. (**A**) Boxplot of *BRCA1* expression in luminal A and B tumors (p value < 0.001, the Wilcoxon rank sum test), (**B**) Scatterplot of *MKI67* and *PCNA* expression levels in luminal A and B tumors, (**C**) Bar graph of luminal A and B tumor numbers in low- or high-*BRCA1* groups (left panel: cutoff = median of *BRCA1* expression levels, Fisher’s exact p value = 0.019, right panel: cutoff = 8th decile of BRCA1 expression levels, Fisher’s exact p value = 0.021).

BRCA1 is known to interact with ERs, which may in turn regulate mammary cell proliferation^38–40^. Thus, we focused on molecular types, luminal A and B, of ER^+^ tumors to ensure the study samples were homogeneous. The GEO dataset GSE45827^41^ containing primary breast tumors with different molecular types allowed us to compare the *BRCA1* expression levels in different types of ER^+^ tumors. The expression level of *BRCA1* was significantly higher in luminal B tumors (Fig. 1A, p value < 0.001, Wilcoxon test). By definition, luminal B tumors have a high level of the proliferative marker Ki-67 and generally grow faster than luminal A tumors. In addition to *MKI67*^42,43^ (gene of Ki-67), *PCNA*^43–45^ (proliferating cell nuclear antigen) is also a breast cancer proliferation marker. In the scatterplot (Fig. 1B) of the expression levels of *MKI67* and *PCNA* in the tumors, luminal B tumors were mostly located in the upper right part. Even though the gene expression level was not always proportional to the protein level, this result appeared consistent with the proliferative behavior of luminal B tumors. To check whether high *BRCA1* expression was significantly associated with tumor proliferation, we calculated the proportions of luminal B tumors in the low- and high-*BRCA1* expression groups. Dataset GSE45827 had 29 luminal A and 30 luminal B tumors. These 59 luminal tumors were divided into low- and high-BRCA1 groups by the median or 8th decile of their *BRCA1* expression levels. On the basis of either cutoff (median or 8th decile), the low-*BRCA1* expression group had a higher proportion of luminal A tumors, and the high-*BRCA1* expression group had a higher proportion of luminal B tumors (Fig. 1C). The high proportion of luminal B tumors in the high *BRCA1* expression group confirmed the association between high *BRCA1* expression and cell proliferation.

If the relatively aggressive luminal B tumor expressed more *BRCA1* transcripts, we expected high *BRCA1* expression patients to have not only high proliferation marker expression but also rapid distant metastasis (DM).

### Distant metastasis-free survival analysis using a “cure model” is the correct method to assess the aggressiveness of tumors

Since a patient may not have an expectation of long-term survival once she develops DM, DMFS is a good endpoint for marking the aggressiveness of tumors. In an attempt to attain homogeneity among study subjects in survival analysis, only early-stage ER^+^ patients who received standard tamoxifen treatment^21^ after surgery were included in the DMFS analysis. As described in “Materials and methods”, only 3 datasets, GSE12093^46^, GSE17705^47^ and GSE45255^48^, were selected from the GEO database. A “cure model,” the logistic-AFT location-scale mixture regression model^49^, was utilized in this study to tackle the nonsusceptibility of DM, which would be ignored by the commonly used Cox proportional hazards regression model^50^ in survival analysis. Cure models have been a popular topic in the statistical literature for decades but are not as widely known in the medical literature^51^. Some satisfactory applications of these models involved studying cures in cancer clinical trials^51,52^ and nonsusceptibilities of alcoholism^53^ and metabolic syndrome^49,54^. Cox’s model^50^ assumes that not only hazard ratios among the strata are proportionally maintained as constants independent of the follow-up time but also, implicitly, all the subjects are susceptible to, or at risk under the study event. However, not every breast cancer patient develops DM after surgery, so the cure model, which considers DMFS as cured, provides an adequate statistical approach for this study.

The *BRCA1* expression level is a continuous variable, and the histogram, which is composed of the *BRCA1* expression levels of the pooled samples from the GEO datasets, is unimodal (Supplement Fig. S1). To compare the differences in the DMFS time of the high- or low-*BRCA1* expression groups, a threshold for *BRCA1* expression is needed. Nine deciles in the histogram representing *BRCA1* expression distribution were used to categorize the patients into high- or low-expression groups. The difference between the Kaplan–Meier overall DMFS curves (step functions) of these two *BRCA1* expression groups defined by a specified decile was then assessed with the significance test by fitting it to the logistic-AFT mixture regression model^49^.

**Figure 2:**
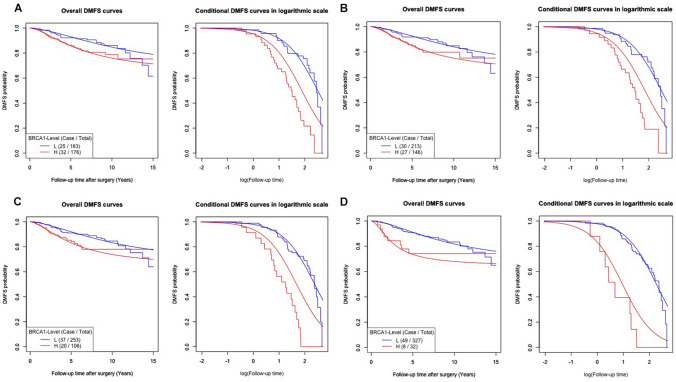
Survival curves fitted by the “Logistic-AFT mixture regression model” at the 5th–7th and 9th deciles. Kaplan–Meier (step function) and mixture regression (smooth curve) estimators of overall and conditional DMFS curves for the DM onset time after surgery stratified by BRCA1 level defined at the cutoff points (**A**) 5th, (**B**) 6th, (**C**) 7th, and (**D**) 9th deciles, and the corresponding p values of the likelihood ratio test (P_LRT_) were 0.024, 0.023, 0.020 and 0.005, respectively. The *BRCA1* high- and low-expression groups are shown in red and in blue, respectively, for each cutoff point.

**Figure 3:**
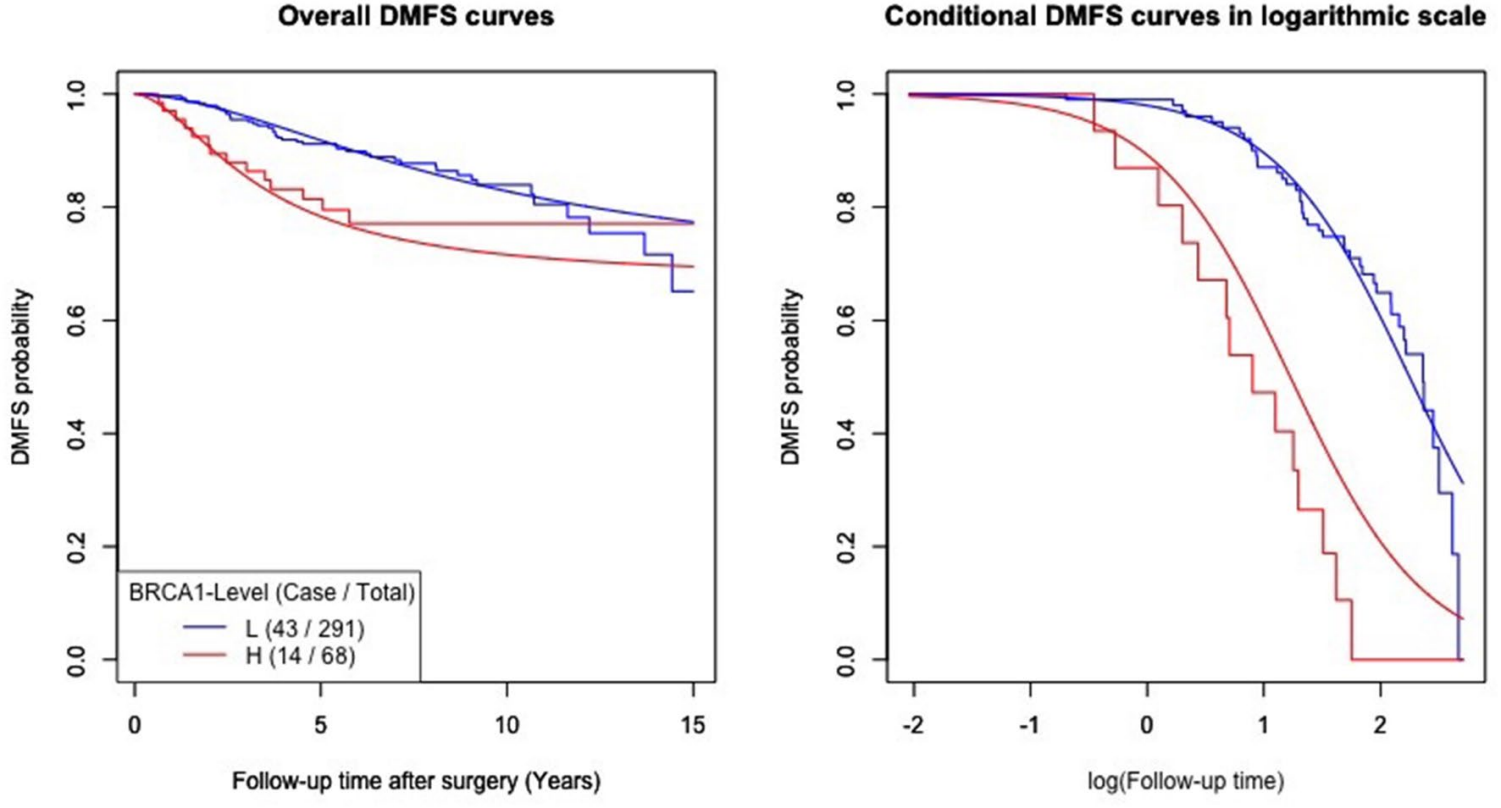
Survival curves fitted by the “Logistic-AFT mixture regression model” at the 8th decile. Kaplan–Meier (step function) and mixture regression (smooth curve) estimators of overall and conditional DMFS curves for the DM onset time after surgery stratified by BRCA1 level defined at the cutoff point 8th decile. The *BRCA1* high- and low-expression groups are shown in red and in blue, respectively. P value of likelihood ratio test (P_LRT_) was 0.004.

The results from fitting the logistic-AFT mixture model^49^ were not significant for each cutoff point ranging from the 1st to 4th deciles (results not shown). The results falling into the 5th to 7th and the 9th deciles are reported in Fig. 2A–D and Table 1, and those for the cutoff point at the 8th decile (the best-fitting model) are displayed in Fig. 3 and Table 1. In each of these figures, the left panel indicates the overall DMFS curves, and the right panel indicates the conditional DMFS curves according to a logarithmic time scale. The former displays the DMFS probability, including the lifetime DMFS probability in the long run, based on all patients at each follow-up time after surgery, and the latter estimates the distribution of time to DM, including the median onset time after surgery, of the susceptible patients only. Conceptually, the susceptible patients consisted of all the patients observed with DM and the other patients who never had an onset weighted by their individual probabilities of later onset of DM after the follow-up time elapsed.

**Table 1:** Analysis of the log-logistic-AFT mixture regression model stratified by BRCA1-level with different cutoff points.

Cutoff decile	Covariate	Logistic regression submodel		AFT submodel (log-logistic event time distribution)					Mixture model p value^b^
				Location regression part			Scale regression part		
		Estimate	95% CI	Estimate	95% CI	p value^a^	Estimate	95% CI	
50%	Intercept	− 0.562	− 1.558, 0.434	2.506	1.749, 3.263		− 0.468	− 0.805, − 0.131	0.024
	BRCA1-H			− 0.620	− 1.147, − 0.093	0.021			
60%	Intercept	− 0.539	− 1.546, 0.468	2.475	1.718, 3.232		− 0.466	− 0.801, − 0.131	0.023
	BRCA1-H			− 0.632	− 1.163, − 0.101	0.020			
70%	Intercept	− 0.587	− 1.518, 0.344	2.384	1.673, 3.095		− 0.479	− 0.804, − 0.154	0.020
	BRCA1-H			− 0.705	− 1.285, − 0.125	0.017			
80%	Intercept	− 0.715	− 1.319, − 0.039	2.249	1.706, 2.792		− 0.545	− 0.843, − 0.247	0.004
	BRCA1-H			− 1.022	− 1.667, − 0.377	0.002			
90%	Intercept	− 0.598	− 1.349, 0.153	2.271	1.683, 2.859		− 0.510	− 0.800, 0.220	0.005
	BRCA1-H			− 1.304	− 2.131, 0.477	0.002			

CI, confidence interval; BRCA1-H, high BRCA1; LRT, likelihood ratio test.

p value^a^ of Wald’s statistic for testing the location parameter corresponding to BRCA1-H.

p value^b^ of the LRT for testing the significance of the fitted mixture model.

### High ***BRCA1*** gene expression is associated with rapid distant metastasis in ER^+^ early-stage tamoxifen-treated breast cancers

The corresponding overall DMFS curves of the high-*BRCA1* expression groups (red step functions in the left panels of Figs. 2C,D and 3) categorized by higher deciles (such as 70% to 90%) flattened off. As shown in Fig. 3, the model-predicted curves (smooth curve) fit closely to the Kaplan–Meier DMFS curves, which indicated that the cure model could correctly predict the survival of patients in the pooled GEO datasets, with a cured proportion.

Varying from the 5th to 9th deciles, all the corresponding logistic regression submodels did not include the high *BRCA1* expression variable. They indicated that the DMFS probabilities of the low- and high-*BRCA1* expression groups defined by each decile were similar, as shown by the smooth curves in Figs. 2 and 3. The significant p values were a result of the likelihood ratio test (LRT) for the mixture models and confirmed the *BRCA1* gene expression dependency with increasing *BRCA1* expression levels. This trend was revealed by the statistical significance of high *BRCA1* expression in the “Location Regression Part” in Table 1, together with the well-separated low- and high-*BRCA1* expression (smooth) conditional DMFS curves in Figs. 2 and 3. The separation due to a negative estimate of the location parameter resulted in a shorter onset time to DM in the high-*BRCA1* expression group than in the lower expression group. On the other hand, each of the “Scale Regression Parts” in Table 1 did not include high *BRCA1* expression, so the dispersions of the low- and high-*BRCA1* expression conditional DMFS curves were similar.

Consequently, in Table 1, the p value of the LRT corresponding to the 8th decile is the smallest (0.004, still significant even after adjustment for multiple comparisons) among the LRT p values for all deciles. This suggests that the best-fitting model is based on the 8th decile (of expression level 6.76) as the cutoff; in fact, the optimal model selection was also confirmed by the minimum Akaike information criterion (AIC, the results are not shown). The decreasing trend of the AIC values (results not shown) among increasing cutoff deciles implies that the possibility of overfitting the model by using this optimal cutoff point rather than other points was low. As shown in Fig. 3, this cutoff point sharply contrasts with the difference for the two Kaplan–Meier DMFS curves depicting the high and low levels of *BRCA*1 expression.

The logistic regression submodel (Table 1, 80%) did not include the high-*BRCA1* expression variable, with an estimated intercept of − 0.715, indicating that the estimated common lifetime probability at both expression levels was 0.672. That is, 67.2% of the early-stage ER^+^ tamoxifen-treated breast cancer patients were free of DM during the 15 years of follow-up, regardless of their *BRCA1* expression level. It also readily explained why the Kaplan–Meier overall DMFS curves depicting the *BRCA1* high- (red step function) and low- (blue step function) expression groups in the left panel of Fig. 3 crossed at similar values of the DMFS proportion right before the 15th year of follow-up. The location parameter in the AFT submodel, however, includes a high-*BRCA1* expression variable with a negative parameter estimate of − 1.022 (p = 0.002 of the Wald test, Table 1, 80%). This indicated that *BRCA1* had an impact on the time to DM after surgery, such that the median DM time (3.41 years) for susceptible patients in the high-*BRCA1* expression group was significantly earlier than that (9.75 years) of the low expression group. The left panel of Fig. 3 reveals that the Kaplan–Meier curve of the high *BRCA1* expression group (red step function) dropped sharply and then flattened off afterward. This drop took place because DM in this group continually occurred in the first 5.77 years. Therefore, the rest of the patients appeared to be “cured” toward the end of the 15-year follow-up period. Nevertheless, this phenomenon was not observed in the low *BRCA1* expression group because DM occurred at a steady rate during the 15 years of follow-up.

### The association between high *BRCA1* gene expression and rapid distant metastasis is consistently observed in different datasets

Tamoxifen, through its interactions with the estrogen receptor, can inhibit the proliferative function of ER signaling. Nevertheless, some patients have intrinsic resistance to tamoxifen, and consequently, they develop recurrence very early^55^. If the high-*BRCA1* tamoxifen-treated group in the previous section was heavily populated with patients who had intrinsic tamoxifen resistance, their average time to DM would be rather short. This putative mechanism can be tested by comparing the treated group to the tamoxifen untreated patients whose DMFS times should be irrelevant to tamoxifen resistance. Untreated patients from two breast cancer datasets, GSE7390^56^ and GSE2034^57^, were thus added to the survival analysis. Dataset GSE7390 had a very similar DMFS pattern to the aforementioned treatment group, but GSE2034 had a high proportion of poorly differentiated tumors^58^ with a poorer DMFS curve displaying a biphasic pattern (Supplement Fig. S2). Because our study is not from a randomized control trial, to be cautious, these two untreated datasets were compared to the treated cohort separately due to their different survival patterns.

**Figure 4:**
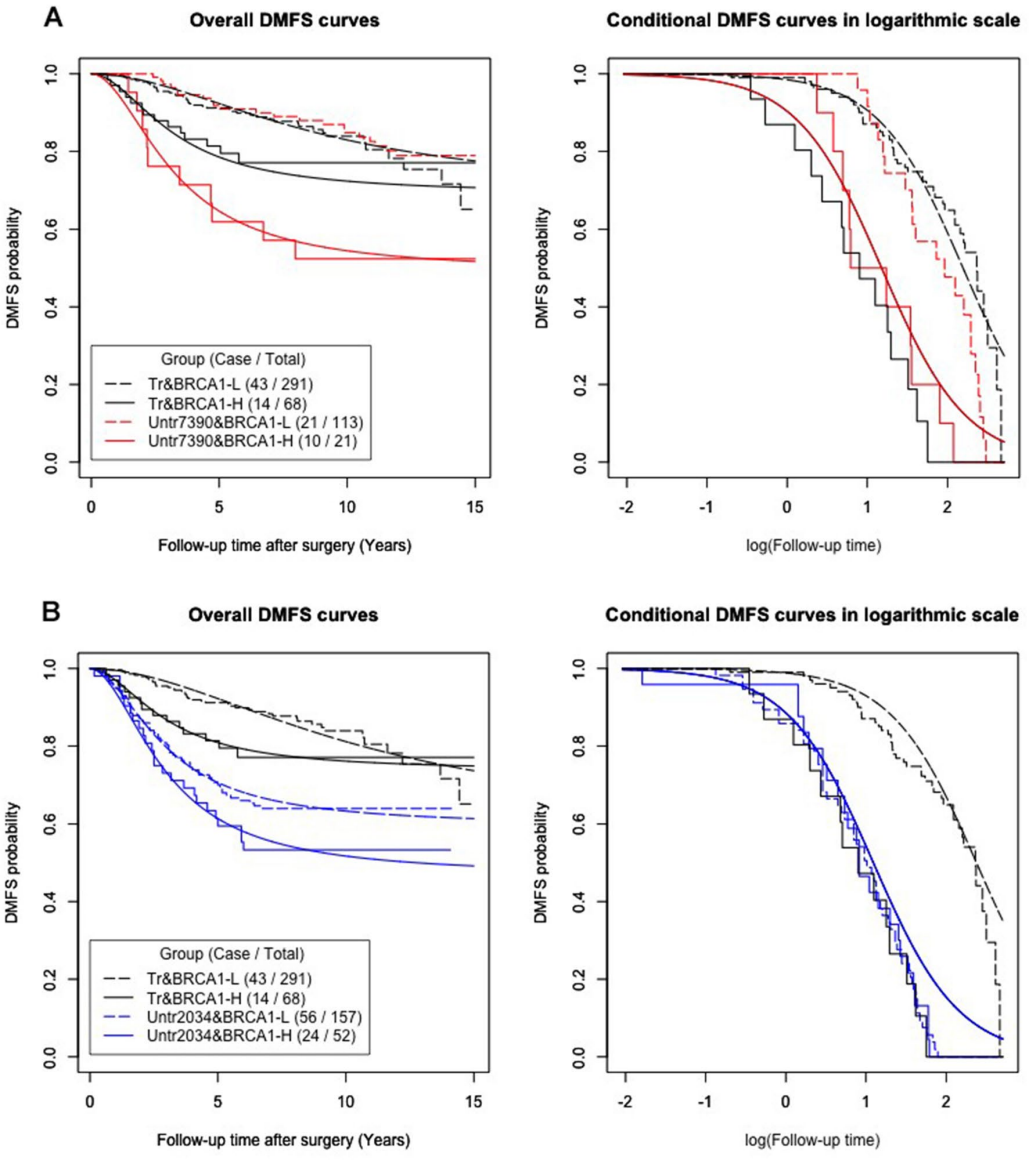
Logistic AFT mixture regression models for tamoxifen-treated and untreated patients. The chosen model was based on *BRCA1* cutoff 8th decile. Survival curves for the tamoxifen-treated cohort, untreated GSE7390 cohort and untreated GSE2034 cohort are shown in black, red and blue, respectively. High- and low-*BRCA1* expression subgroups are shown with solid and dashed lines, respectively. (**A**) Treated group and untreated GSE7390. In the overall DMFS plot, the two low-*BRCA1* groups (red and black dashed lines) were fitted as the same group by the model. In the conditional DMFS plot, the two low-*BRCA1* groups were fitted as the same group by the model, as were the two high-*BRCA1* groups. (**B**) Treated group and untreated GSE2034. In the conditional DMFS plot in the right panel, both BRCA1 expression groups of GSE2034 were fitted to be the same as those of the high BRCA1-treated patients.

*BRCA1* cutoffs from the 1st to 5th deciles did not show any significant difference (results not shown) in each separate DMFS analysis. The LRT p values for both comparison sets (treated vs. GSE7390 and treated vs. GSE2034), by using the *BRCA1* cutoff from 60 to 90%, were significant and showed an increasing trend in survival difference (results not shown). The DMFS curves in Fig. 4 were defined by the same cutoff at 80% of the *BRCA1* expression level, as in the preceding analysis based only on tamoxifen-treated patients. In each separate DMFS analysis, the cutoff point at 80% corresponded to the (almost) best-fitting model. The model-fitting results are shown in Supplementary Table S3A,B.

Figures 4A,B demonstrate the quite different survival curves between the two untreated datasets. Most noticeably, in patients with high *BRCA1* expression, the treated (black solid line in Fig. 4A,B) patients had a better overall DMFS curve than the untreated (red solid line in Fig. 4A and blue solid line in Fig. 4B) patients. Qualitatively speaking, tamoxifen treatment improved the lifetime DMFS probabilities (i.e., the cured fractions) of the high-*BRCA1* expression patients by approximately 20% in comparing the flattened probability levels on the black *versus* colored smooth curves. In contrast, among the high-*BRCA1* patients susceptible to DM, the conditional DMFS curves of untreated patients in both GSE7390 (red solid line in the right panel of Fig. 4A) and GSE2034 (blue solid line in the right panel of Fig. 4B) were close to that of the treated patients (black solid line). Thus, the major treatment efficacy of tamoxifen emerged with respect to the probability of achieving lifetime DMFS.

On the other hand, considering the low *BRCA1* expression patients, the overall DMFS curves (Fig. 4A, left panel) and the conditional DMFS curves (Fig. 4A, right panel) of both the treated (black dashed line) and untreated GSE7390 (red dashed line) groups almost overlapped. In contrast, the overall DMFS curves (Fig. 4B, left panel) and the conditional DMFS curves (Fig. 4B, right panel) of both the low-*BRCA1* expression patients in both the treated (black dashed line) and untreated GSE2034 (blue dashed line) groups were well separated (Fig. 4B). These differences could be explained by the higher incidence of poorly differentiated tumors in dataset GSE2034, but further information for verification is lacking.

Regardless of the tamoxifen treatment, all three high-*BRCA1* expression groups showed not only biphasic DMFS patterns but also poorer DMFS than their corresponding low-*BRCA1* groups. In other words, the associations between high *BRCA1* expression and aggressive tumor behavior (poor DMFS) were consistently revealed within the tamoxifen-treated patients and within each of two other datasets of patients who did not receive tamoxifen treatment. These consistent results indicated that the association between high *BRCA1* expression and poor DMFS was not merely due to intrinsic tamoxifen resistance.

Because tamoxifen treatment improved the overall DMFS of high *BRCA1* expression patients, the effect of *BRCA1* expression on the time to DM was likely mediated by ER signaling. Although ER signaling is known to trigger proliferation, it is necessary to show that high *BRCA1* expression is associated with proliferation.

**Figure 5:**
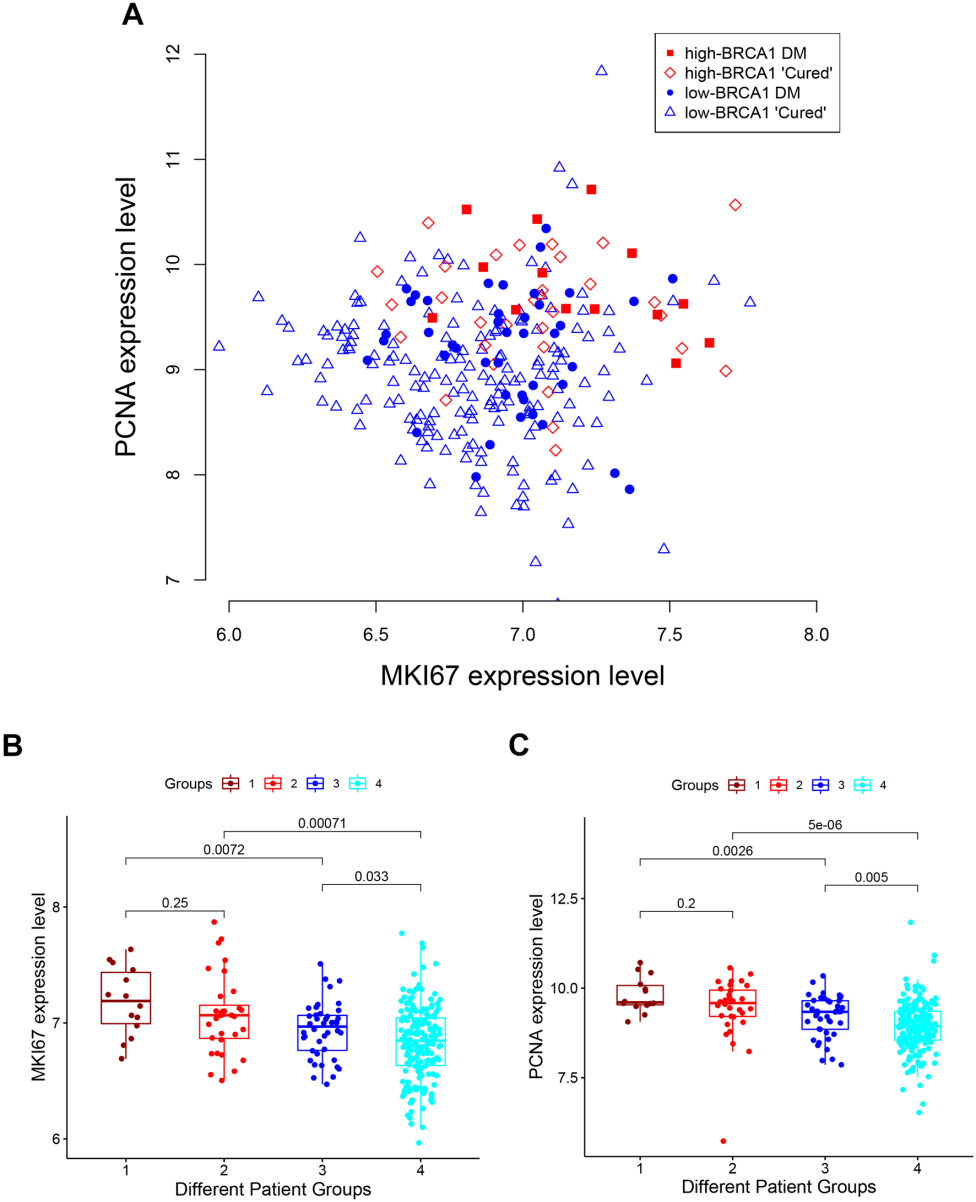
*MKI67* and *PCNA* expression in 359 tamoxifen-treated patients. (**A**) Scatterplot of *MKI67* and *PCNA* expression levels for 4 groups of patients. (**B**) Boxplot of *MKI67* expression in 4 groups of patients. (**C**) Boxplot of *PCNA* expression in 4 groups of patients. 1 = High *BRCA1* patients with DM, 2 = High *BRCA1* “cured” patients (i.e., without DM and follow-up longer than 5.77 years), 3 = Low *BRCA1* patients with DM, 4 = Low BRCA1 “cured” patients (*i.e.,* without DM and follow-up longer than 5.77 years).

The potential of proliferation, which was represented by the expression of *MKI67* and *PCNA* genes in a scatterplot, successfully distinguished luminal A and B cells (Fig. 1B). A similar strategy can be used to present the potential proliferation of those tumors in survival analysis. The other two types of information, namely, *BRCA1* expression level and survival behavior, can be overlaid on this scatterplot by using different colors and types of symbols (Fig. 5A). Thus, the relationship between these characteristics of each patient can be tracked. All DM in the high *BRCA1* expression group occurred before 5.77 years. Those who did not develop DM but were followed-up shorter than 5.77 years might offer ambiguous information because they would have later onset or be eventually cured. Thus, they were excluded from the subsequent analysis. The high- and low-*BRCA1* expression groups could be further separated into DM (shown by red solid squares and blue solid circles, respectively) and “cured” (shown by red empty diamonds and blue triangles, respectively) subgroups. The definition of “cured” here was that during follow-up a patient did not experience any event of DM for longer than 5.77 years.

Tumors with high B*RCA1* expression were located in the upper right part and thus showed higher proliferation activity than tumors with low BRCA1 expression. Furthermore, in the DM subgroups, tumors with high *BRCA1* expression appeared more aggressive than tumors with low *BRCA1* expression not only because they were located in the right upper part of the scatterplot but also because they developed DM much earlier. To further illustrate the significant difference between the high- and low-*BRCA1* groups, the levels of *MKI67* (Fig. 5B) and *PCNA* (Fig. 5C) expression and the p values were compared.

The expression levels of both *MKI67* (Fig. 5B) and *PCNA* (Fig. 5C) in the rapid DM high-*BRCA1* tumors (Group 1 in the Figures) were significantly higher than those in the gradually metastatic low-*BRCA1* tumors (Group 3, P values = 0.0072 and 0.0026, respectively). These findings indicated that rapid DM in high-*BRCA1* patients was associated with increased proliferation activity. The associations among “high *BRCA1* expression”, “potential for proliferation” and “early metastasis” implied that rapid DM was likely caused by ER signaling proliferation.

## Discussion

The logistic-AFT mixture cure model provides a sophisticated statistical approach to explore consistent findings for elucidating the controversial issues of *BRCA1* gene expression and aggressive tumor behavior. Despite the limitation of a pooled data analysis, there were 3 cohesive observations in this study. First, high *BRCA1* expression was associated with aggressive tumors, such as luminal B in ER^+^ tumors. Second, the high *BRCA1* expression group displayed a biphasic overall DMFS curve, which implied that there were susceptible patients (metastasized rapidly) and nonsusceptible patients (eventually cured). The association of high *BRCA1* expression and a high risk of DM ended previous conflicting reports on the relationship between *BRCA1* expression and the aggressiveness of tumors. Third, the aggressive tumor behavior of patients with high *BRCA1* expression could be improved by approximately 20% with tamoxifen treatment. These findings shed light on the possible mechanism of the reported association.

**Figure 6:**
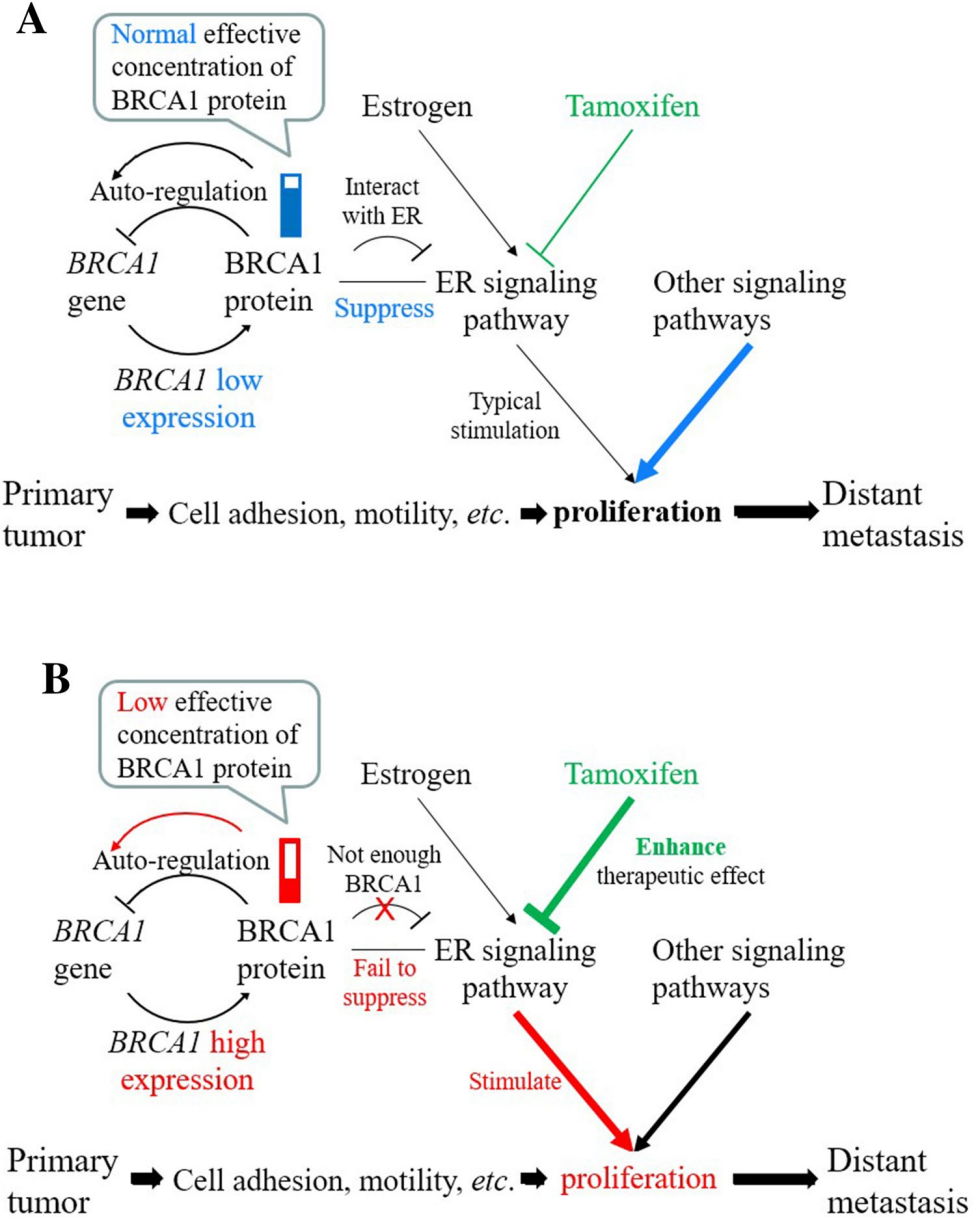
A comprehensive interpretation to link our observations and literature reports. This figure was drawn by Microsoft PowerPoint version 14.6.2 (https://www.microsoft.com/en-us/microsoft-365). (**A**) The pathway for the primary tumor to develop DM under the condition of sufficient BRCA1 protein (leading to low *BRCA1* expression via autoregulation). (**B**) The pathway for the primary tumor to develop DM under the condition of low effective BRCA1 protein concentration (leading to high *BRCA1* expression via autoregulation).

A testable hypothesis was proposed to connect these facts. The development of DM is a complicated process. As shown in the bottom of Fig. 6, many processes are necessary, but each of them may not be sufficient to cause DM. For example, tumor cells need to detach from the primary site and migrate into the blood or lymphatic vessels before these circulating tumor cells land at a distant site^59^. After landing, the metastasized tumor cells have to proliferate to a size large enough so that they can be detected by imaging instruments. This argument is supported by the fact that aggressive ER^+^ tumors, such as the luminal B subtype, express higher proliferation markers, such as Ki-67^60^ protein. This proliferation behavior is also reflected at the level of gene expression for *MKI67* (encodes KI-67 protein) and *PCNA* genes (Fig. 1B). Because tumors with high *BRCA1* expression also have high expression of these two proliferation marker genes (Figs. 1B and 5), *BRCA1* expression-dependent proliferation is likely associated with the early occurrence of DM (Figs. 3 and 4). This association was observed by DMFS analyses over different datasets regardless of tamoxifen treatment.

As shown in Fig. 6, many pathways, such as ER and other signaling pathways, may stimulate proliferation. However, *BRCA1* expression-dependent proliferation is likely triggered by the ER signaling pathway. This is because tamoxifen, an inhibitor of the ER signaling pathway, may improve the DMFS fraction by approximately 20% (Fig. 4A,B). This proposed mechanism was supported by the fact that the BRCA1 protein interacts directly or indirectly with the ER and suppresses ER signaling^12,38^. Thus, it was reasonable that the DMFS of our ER^−^ tumors had no obvious association with BRCA1 gene expression status (data not shown) because ER^−^ tumors lacked estrogen receptor to interact with BRCA1 protein.

In Fig. 6A, fully active BRCA1 protein was presumably sufficient to suppress the proliferation induced by ER activity. Therefore, the ER signaling pathway had little impact on tumor proliferation. The DM of tumors depends mostly on other signaling pathways (thick blue arrow) that stimulate tumor growth. If, however, BRCA1 protein function was decreased (Fig. 6B) either qualitatively (*e.g.,* protein activity loss) or quantitatively (*e.g.,* decreasing the number of effective proteins in the nucleus), ER signaling proliferation may escape suppression of the BRCA1 protein. A failure to suppress ER signaling proliferation may make a patient susceptible to fast proliferation (thick red arrow) and consequently early DM. Therefore, the effect of tamoxifen treatment (antiestrogen treatment) was enhanced in these patients and could improve patient survival by up to 20%, as shown in Fig. 4A,B.

The BRCA1 protein autoregulates its own gene^35^ and this bridges the gap between high *BRCA1* expression and ER signaling proliferation. As described in the introduction, loss of BRCA1 protein staining in the nucleus was often observed in sporadic breast cancers, and this phenomenon was associated with high-grade and poor survival. As shown in Fig. 6B, sensing a loss of functional BRCA1 protein in the nucleus, a cell might try to amend the disorder by increasing *BRCA1* gene transcription. If the increased *BRCA1* transcripts failed to increase the BRCA1 protein level, ER signaling could not be fully suppressed. As a result, tumors with high *BRCA1* expression may lead to rapid DM in early ER^+^ patients.

Most germline BRCA1 mutations lead to ER^−^ tumors^9,10^. Similarly, somatic *BRCA1*-mutated tumors can mostly be ER^−^ because defective BRCA1 protein interferes with *ESR1* transcription^8^. In contrast, approximately 20% of sporadic ER^+^ breast tumors, which had high *BRCA1* expression in this study, were significantly associated with poor patient survival through reduced BRCA1 activity and uncontrolled ER signaling. Understanding the plausible mechanisms of these tumors is useful for the precise treatment of patients. This working hypothesis explains not only the short time to DM for high *BRCA1* expression patients but also the similar lifetime DMFS probabilities in both high and low *BRCA1* expression patients receiving tamoxifen treatment (Fig. 3). This is because only proliferation, not the chance of tumor landing, is affected by the loss of BRCA1 function. The proliferation of landed tumor cells might gradually be triggered by other types of signaling pathways in low *BRCA1* expression patients. Moreover, in addition to mutations in the coding region, other factors changing the effective concentration of BRCA1 protein might represent another type of BRCAness. On the basis of this working hypothesis, novel therapy for tumors with BRCAness^61^, such as PARP inhibitors, might be considered in treating patients with high *BRCA1* expression.

## Materials and methods

### Study samples

Microarray data used in this study were all from the GEO repository^37^. To examine *BRCA1* gene expression in molecular subtypes of ER^+^ breast tumors, we chose dataset GSE45827^41^, which is composed of 130 primary invasive breast cancers (41 Triple Negative, 30 Her2, 29 Luminal A and 30 Luminal B tumors) as well as 11 normal tissues and 14 cell lines, using Affymetrix HG U133 plus 2.0 as the study platform. Because this study focused on ER^+^ tumors, only the *BRCA1* expression levels of normal breast tissue and luminal A and luminal B tumors from GSE45827 were compared.

Two criteria were considered in selecting the datasets for survival analysis: (1) datasets that tracked the survival time with related clinical data; (2) datasets with an identical technology platform for microarray gene expression data. Ten breast cancer datasets with survival records, which used the U133A platform for expression analysis, were thus selected from the GEO database (see clinical information in Supplementary Table S1). However, because DMFS could be more directly associated with tumor behavior, the DMFS time was used as the endpoint of the patients’ prognosis so that only those datasets with the DMFS time were selected for our study. To make our study samples homogenous, the following inclusion criteria were further developed to select ER^+^ patients for early detection and treatment: (1) the tumor was localized at its original locus, (2) the lymph nodes were free of tumor, and (3) the tumor size was not larger than 5 cm. Study subjects were required to have an estrogen receptor status and adjuvant treatment information. After thoroughly examining patients’ clinical information, 359 patients in three GEO datasets (GSE12093^46^, GSE17705^47^ and GSE45255^48^) were selected as the treated group in our survival analysis (see Supplementary Table S2).

Another two untreated tamoxifen patient datasets, GSE7390^56^ and GSE2034^57^ (see Supplementary Table S1), were further added to the survival analysis section. They were composed of 286 LN- breast cancer samples (209 ER^+^ and 77 ER^−^) and 198 LN- breast cancer samples (134 ER^+^ and 64 ER^−^). Only ER^+^ samples from these two datasets were included in the analysis.

### Microarray data analysis

The microarray data were normalized by using the Bioconductor R “affy” package^62^. To minimize the false-negative and false-positive rates, the parameters were set up based on Choe’s^63^ study. Namely, bgcorrect.method = “mas”, pmcorrect.method = “pmonly”, normalize.method = “quantiles”, and summary.method = “medianpolish” were set up and input into the “affy” package’s “expresso” function. All samples of GSE45827 data (on the HG-U133 plus 2.0 platform) were normalized. A total of 1057 samples from five chosen GEO datasets, GSE12093, GSE17705, GSE45255, GSE2034 and GSE7390 (on the HG-U133A platform), were normalized together. After normalization, eligible study samples satisfying the aforementioned inclusion criteria were selected for further analysis.

The R package “jetset”^64^ was used to select the most representative probe set from multiple probe sets of a gene in the Affymetrix microarray. This program computes “jetscores” and uses the probe set of the highest score to designate a gene for further analysis. In the Affymetrix HG U133 series, the *BRCA1* gene has two corresponding probe sets, 204531_s_at and 211851_x_at, with jetscores of 0.278 and 0.029, respectively. Therefore, 204531_s_at was substituted for *BRCA1* gene expression in our analysis.

With the same procedures, 212023_s_at and 201202_at were used to represent the MKI67 and PCNA genes, respectively.

### Statistical methods

#### The Kruskal–Wallis test

To compare the differences in the *BRCA1* gene expression levels among several groups, the nonparametric Kruskal–Wallis test was used to test the null hypothesis that these independent samples came from the same population distribution. Upon rejection of the null hypothesis using the Kruskal–Wallis test, we conducted multiple pairwise comparisons to test the distributional difference between any two groups based on the Wilcoxon rank sum statistic.

#### Logistic-accelerated failure time (AFT) mixture regression model

We employed the logistic-AFT mixture regression model^49,53^ to compare two Kaplan–Meier curves^65^ representing the DMFS time between high-level and low-level *BRCA1* expression, which was defined by a specified cutoff point of BRCA1 expression values, with only right censored data. A patient was either an event case with DM or right censored with a follow-up DMFS time. Fitting a patient’s indicator covariate representing high *BRCA1* expression into the mixture model simultaneously estimated her lifetime risk probability of DM and the distribution of her DMFS time if she would be susceptible to DM. A brief introduction to the mixture cure model is as follows:

Let *T* be the duration in years from the surgery to DM in breast cancer, and the survival distribution at *t* years Pr(*T* > *t*) indicates the probability that a patient’s cancer will not metastasize for *t* years. The patient’s eventual susceptibility to DM is represented by the binary indicator *D*, where *D* = 1 indicates that the patient is susceptible and D = 0 indicates that the patient is not susceptible. The probability that a patient with a risk factor *Z*, such as an indicator of high *BRCA1* expression, will not metastasize after *t* years is expressed as:

$$ \Pr \left( {T\, > \,t|Z} \right)\, = \,\Pr \left( {D\, = \,1|Z} \right)\Pr \left( {T\, > \,t|D\, = \,1,Z} \right)\, + \,\Pr \left( {D\, = \,0|Z} \right), $$

where, for a given *Z*, Pr(*D* = 0|*Z*) is the lifetime DMFS probability and Pr(*T* > *t*|*D* = 1, *Z*) is the conditional survival function at *t* years for a susceptible patient.

The logistic-AFT mixture regression model^49^ contains both a logistic regression submodel

$$ln\left(\frac{\mathrm{Pr}(D=1|Z)}{\mathrm{Pr}(D=0|Z)}\right)= {\beta }^{\mathrm{^{\prime}}}Z,$$

and an AFT location-scale regression submodel

$$lnT\left(Z\right)= {\gamma }^{\mathrm{^{\prime}}}Z+exp\left({\alpha }^{\mathrm{^{\prime}}}Z\right)\varepsilon .$$

Parameters $\beta$, $\gamma$, and $\alpha$ are in the logistic, location, and scale regression segments, respectively, and can be simultaneously estimated using the R package “MixtureRegLTIC”^66^. The log-transformed time to metastasis in the AFT model is supposed to have a linear relationship with the risk factor *Z* via the parameters γ. The logarithmic time scale with a multiplicative effect of “acceleration” by the factor $exp\left({\gamma }^{^{\prime}}Z\right)$ can assist in finding initial estimates of $\gamma$ and $\alpha$ from the Kaplan–Meier curves^65^. For a specific value of *Z*, the logistic submodel provides a parametric estimator for the lifetime DMFS probability Pr(*D* = 0|*Z*), which can also be estimated nonparametrically by the tail of the Kaplan–Meier overall DMFS curve. In contrast, the AFT submodel formulates the parametric distribution of the DMFS time on a logarithmic scale for a susceptible patient, while the Kaplan–Meier conditional DMFS curve is displayed as its nonparametric counterpart.

Since the log-logistic distribution was much better fitted to our pooled GSE breast cancer datasets for the AFT submodel than the other generalized gamma distribution models available in the MixtureRegLTIC, we assumed that *T* of a susceptible patient follows a log-logistic distribution in the AFT location-scale regression submodel; that is, $\varepsilon$ is a logistic error term with the density $f\left(\varepsilon \right)={e}^{\varepsilon }/{\left(1+{e}^{\varepsilon }\right)}^{2}$.

According to the minimum Akaike information criterion (AIC), we determined the best model selection of the covariate to be included in the suitable regression parts of the logistic-AFT location-scale mixture regression model. To check the adequacy of the fitted model for high and low BRCA1 expression, we plotted the overall survival curves Pr(*T* > *t*|*Z*) and the conditional curves Pr(*T* > *t*|*D* = 1, *Z*) by superimposing on the corresponding Kaplan–Meier overall and conditional curves to visualize the goodness-of-fit for the best-fitting mixture model. The resultant p value of the likelihood ratio test in the best-fitting mixture model was used to evaluate the significance of the difference between the Kaplan–Meier overall DMFS curves.

## Supplementary Information

Supplementary Information.

## Data Availability

Data used in this study are all from free public resources, NCBI, GEO genomics repository.
