# Supplementary material for: High BRCA1 gene expression increases the risk of early distant metastasis in ER+ breast cancers
Source: Sci Rep. 2022 Jan 7;12:77. doi: 10.1038/s41598-021-03471-w (PMC8741892; doi:10.1038/s41598-021-03471-w)
Supplement: Supplementary file 1 — Supplementary Information. [file 41598_2021_3471_MOESM1_ESM.pdf]

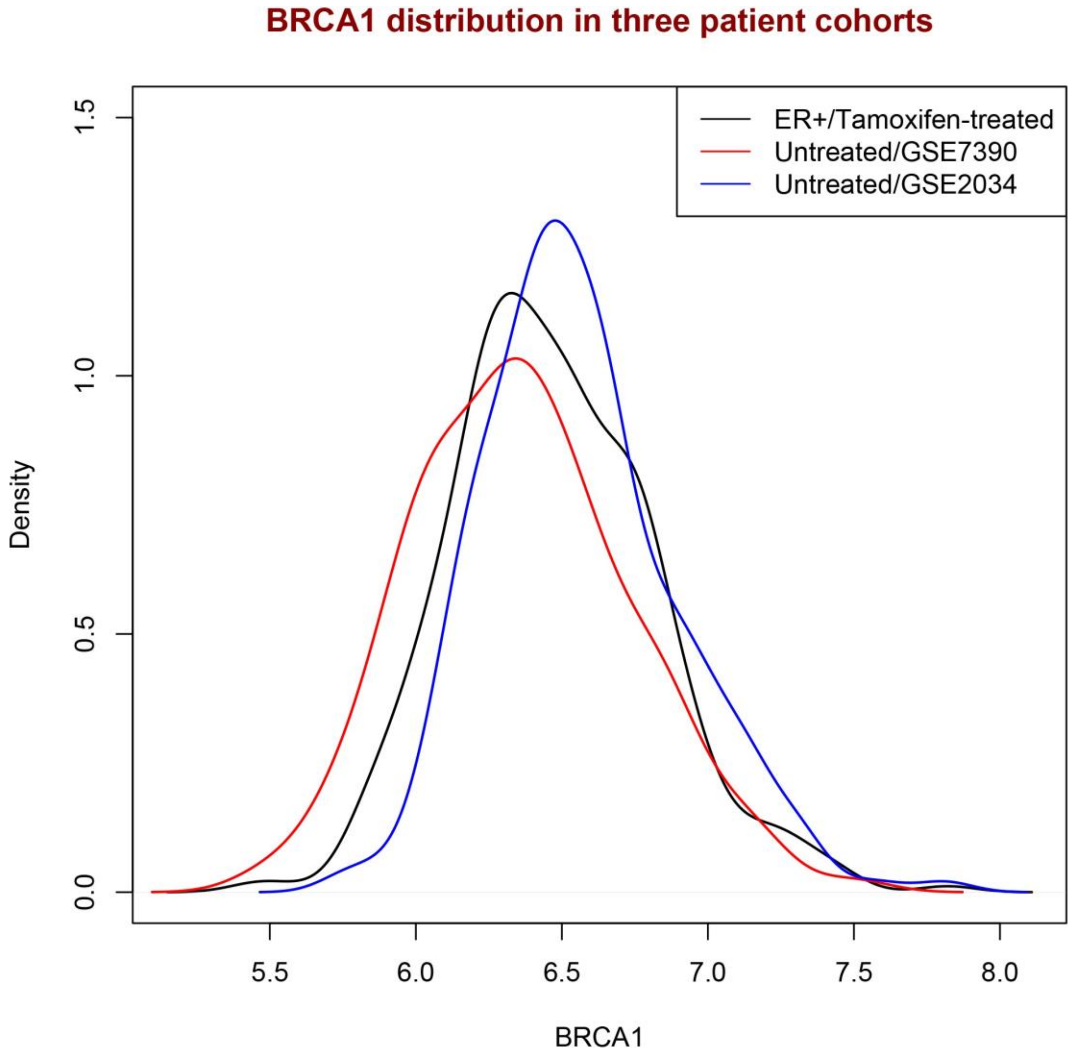

Supplementary Figure S1: Distribution plot showing BRCA1 gene expression for 3 patient cohorts. The curve illustrating 359 tamoxifen-treated patients colored in black, untreated GSE7390 patients in red, and untreated GSE2034 patients in blue.

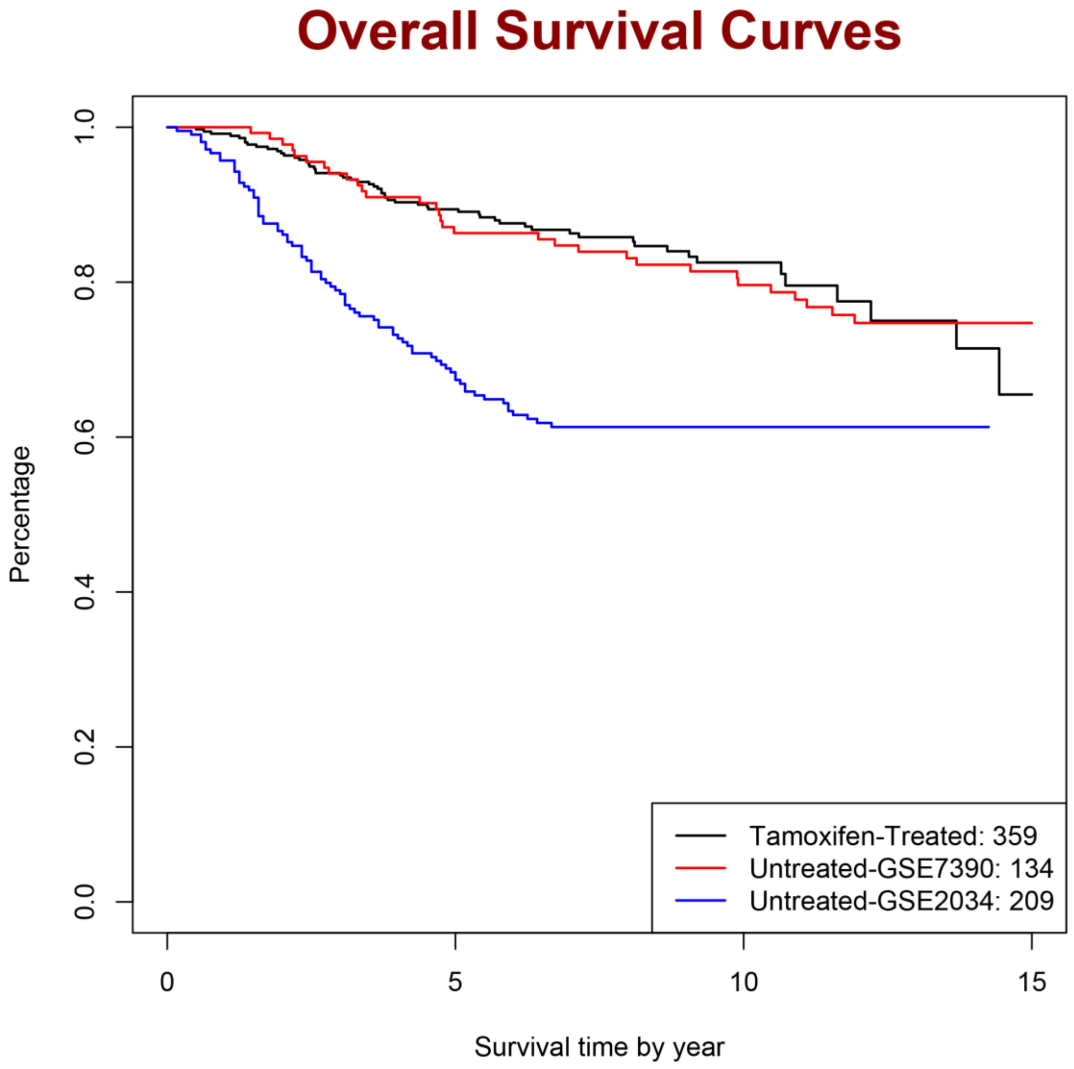

Supplementary Figure S2: The overall distant metastasis-free survival curves for 3 patient cohorts. The curve depicting 359 tamoxifen-treated patients colored black, untreated GSE7390 patients red, and untreated GSE2034 patients blue.

Supplementary Table S1: Clinical information taken from 10 original datasets with survival records

**GSE-ID**	**GSE12093**	**GSE17705**	**GSE45255**	**GSE6532**	**GSE2034**	**GSE7390**	**GSE4922***	**GSE11121**	**GSE1456**	**GSE5327**
Original number	136	298	139	327	286	198	289	200	159	58
Source	Slovenia, Italy, Germany, US	AUS, IGR, OXF	Singapore, Belgium, Oxford	KIT, KIU, OXFT, OXFU	San Diego, CA,US	TRANSBIG	Singapore	Mainz, Germany	Sweden	Chicago, Illinois
Size (cm)<br>Range<br>Mean	NA	NA	1-8.5<br>2.896	0-7<br>2.23	NA	0.6-5<br>2.18	NA	0.1-6<br>2.07	NA	NA
Lymph node<br>Positive<br>Negative<br>NA	0<br>136<br>0	112<br>175<br>11	45<br>94<br>0	193<br>55<br>79	0<br>286<br>0	0<br>198<br>0	NA	0<br>200<br>0	NA	29<br>29<br>0
ER<br>Positive<br>Negative<br>NA	136<br>0<br>0	175<br>0<br>0	89<br>48<br>2	200<br>45<br>82	209<br>77<br>0	134<br>64<br>0	211<br>34<br>44	NA	NA	NA
Adjuvant therapy	Adjuvant tamoxifen for 5 years	Adjuvant tamoxifen for 5 years	ER+:<br>43 tamoxifen only<br>21 tamoxifen+ Chemotherapy<br>16 Chemotherapy only<br>2 other treatment<br>7 Not available<br>ER-:<br>27 Chemotherapy only<br>5 Tamoxifen only<br>6 Tamoxifen+ Chemotherapy<br>6 no adjuvant treatment<br>4 not available	ER+: Tamoxifen/or not (without records)<br>ER-: 2 patients with PR+ received tamoxifen	Systemically untreated	Systemically untreated	66 ER+: endocrine therapy (tamoxifen?)<br>142 (119 ER+ and 21 ER-): systemically untreated<br>others: not available	Systemically untreated	NA	NA
Survival record	DMFS	DMFS	DFS<br>DMFS<br>DSS	DMFS	DMFS<br>Brain relapse	DMFS	DFS	DMFS	RFS	Lung met free survival<br>Metastasis free survival
Age<br>Range	NA	NA	34-84	24-88	NA	24-60	NA	NA	NA	NA

Mean			55.49	58.09		46.39				
Grade<br>1<br>2<br>3<br>NA	NA	NA	17<br>52<br>67<br>3	68<br>143<br>64<br>52	NA	30<br>83<br>83<br>2	68<br>166<br>55<br>0	29<br>136<br>35<br>0	28<br>58<br>61<br>12	NA
PR<br>Positive<br>Negative<br>NA	NA	NA	72<br>60<br>7	46<br>2<br>279	NA	NA	NA	NA	NA	NA
Her2<br>Positive<br>Negative<br>NA	NA	NA	62<br>69<br>8	NA	NA	NA	NA	NA	NA	NA

Abbreviation:

AUS, Austria; IGR, Institut Gustave Roussy; OXF, Oxford; KIT, Karlsruhe Institute of Technology, Uppsala University; KIU, Guy’s hospital, London, UK; OXFT, John Radcliffe Hospital, Oxford, UK; OXFU, Oxford university, UK

DMFS, distant metastasis free survival. DFS, disease free survival. DSS, disease specific survival. RFS, relapse free survival.

* Samples overlap with GSE45255

Supplementary Table S2: Clinical information of selected samples for survival analysis

GSE-ID (original sample size)	GSE12093[1] (136 samples)	GSE17705[2] (298 samples)	GSE45255[3] (139 samples)
Selected sample number (ER+ with adjuvant tamoxifen treatment, LN-, tumor size <=5cm if record exists)	136	175	48
Source	Slovenia, Italy, Germany, USA	AUS, IGR, OXF	Singapore, Belgium, Oxford
Corresponding date of tumor diagnosis	Institute of Oncology, Ljubljana, Slovenia 1997-1999)<br>National Cancer Institute, Bari, Italy (1990-1998)<br>Technische Universitaet Muenchen, Germany (1992-1999)<br>Cleveland Clinic Foundation, US (1981-2000)<br><br>At least 5 years of follow-up	Sample collection dates: 1978-2002 (not available for individual institutes)<br>AUS (Austria)<br>IGR (Institu Gustave Roussy)<br>OXF (Oxford)	NUH (National University Hospital, Singapore, 2000-2002)<br>IJB (Institut Jules Bordet, Belgium, 1994-2001)<br>JRH (John Radcliffe Hospital, Oxford, 1990-1993)
Adjuvant therapy	Adjuvant tamoxifen alone for 5 years	Adjuvant tamoxifen alone for 5 years	48 patients with Tamoxifen treatment (with or without Chemotherapy)

1. Zhang Y, Sieuwerts AM, McGreevy M, Casey G, Cufer T, Paradiso A, et al. The 76-gene signature defines high-risk patients that benefit from adjuvant tamoxifen therapy. Breast Cancer Res Treat. 2009;116(2):303-9.
2. Symmans WF, Hatzis C, Sotiriou C, Andre F, Peintinger F, Regitnig P, et al. Genomic index of sensitivity to endocrine therapy for breast cancer. J Clin Oncol. 2010;28(27):4111-9.
3. Nagalla S, Chou JW, Willingham MC, Ruiz J, Vaughn JP, Dubey P, et al. Interactions between immunity, proliferation and molecular subtype in breast cancer prognosis. Genome Biol. 2013;14(4):R34

Supplementary Table S3A. Analysis of the logistic-AFT mixture regression model, treated with GSE7390, BRCA1 cutoff at 80%

	Covariates	Estimate	95% CI	p-value
Event Probability	Intercept	-0.805	-1.293, -0.317	
	U7390H	0.843	-0.164,1.850	0.101
Location	Intercept	2.194	1.804, 2.584	
	BRCA1-H	-1.016	-1.486,-0.546	< 0.001
Scale	Intercept	-0.641	-0.876,-0.406	
LRT mixture model				< 0.001

Abbreviations: CI, confidence interval. LRT, likelihood ratio test. U7390H, GSE7390 patients with high BRCA1, BRCA1-H, treated patients with high BRCA1.

Supplementary Table S3B. Analysis of the logistic-AFT mixture regression model, treated with GSE2034, BRCA1 cutoff at 80%

	Covariates	Estimate	95% CI	p-value
Event Probability	Intercept	-1.030	-1.673, -0.387	
	BRCA1-L	0.645	-0.072, 1.362	0.078
	U2034H	1.161	0.271, 2.051	0.01
Location	Intercept	1.091	0.864 1.318	
	TL	1.287	0.942, 1.632	<0.001
Scale	Intercept	-0.628	-0.808, -0.448	
LRT mixture model				< 0.001

Abbreviations: CI, confidence interval; LRT, likelihood ratio test; U2034H, untreated GSE2034 patients with high BRCA1; TL, treated patients with low BRCA1.
